# Complete genome sequence of *Pseudomonas fluorescens* IMGN2 isolated from undisturbed soil

**DOI:** 10.1128/mra.00454-24

**Published:** 2024-08-29

**Authors:** Hyeonmin Ryu, Soomin Kim, Yeongjun Kim, Jeong A. Han, Eun Yu Kim, Ho-Seok Lee

**Affiliations:** 1 Department of Biology, College of Sciences, Kyung Hee University, Seoul, South Korea; 2 Center for Genome Engineering, Institute for Basic Science, Daejeon, South Korea; 3 Gyeonggido Agricultural Research & Extension Services, Hwaseong, South Korea; 4 Division of Natural and Applied Sciences, Duke Kunshan University, Kunshan, Jiangsu, China; 5 Environment Research Center Duke Kunshan University, Kunshan, Jiangsu, China; The University of Arizona, Tucson, Arizona, USA

**Keywords:** *Pseudomonas fluorescens*, biocontrol, plant growth promotion

## Abstract

Here, we report the complete genome sequence of *Pseudomonas fluorescens* IMGN2, highlighting its biocontrol and plant growth-promoting capabilities. The genome analysis reveals genetic features that contribute to its potential in agricultural biotechnology, including genes related to secondary metabolite synthesis and plant-microbe interactions.

## ANNOUNCEMENT

*Pseudomonas fluorescens* is a gram-negative, rod-shaped bacterium known for its biocontrol capabilities and plant growth-promoting properties (1–3). Found predominantly in soil and water ecosystems, it suppresses soil-borne plant pathogens and enhances nutrient uptake (4). Due to its disease resistance and growth promotion qualities, it has been researched for agricultural improvement and environmental sustainability (5). The announcement of *P. fluorescens* IMGN2’s genome aims to improve understanding of its genetic foundation and serve as a resource for comparative genomics studies.

The organism was isolated from soil collected from a depth of 10–15 cm undisturbed land at 37.9281°N, 126.8162°E, Heojunmyo, Republic of Korea. Approximately 25 g of soil was sampled and diluted to 1/100 in 225 mL of distilled water (DW). The mixture was shaken at 150 rpm for 3 h. Afterward, the solution was further diluted to 1/1,000, and 100 µL was spread onto Tryptic Soy Agar (TSA). The plates were incubated for 16 h at 37°C in dark conditions and streaked twice to obtain a pure colony.

Genomic DNA was extracted from a single colony picked from the TSA plate using a Maxwell RSC Tissue DNA kit. The DNA was divided for different sequencing methods. One portion was sheared with the Megaruptor 3 (Diagenode) and purified using AMPure PB magnetic beads (Pacific Biosciences). The PacBio sequencing library was prepared using the PacBio SMRTbell prep kit 3.0. Subsequently, HiFi sequencing was performed on the PacBio Sequel II, yielding a total of 1,338,034,271 base pairs from 144,540 reads, with a coverage of approximately 199×. The mean length of HiFi reads was 9,257 base pairs, with an N50 value of 9,828 base pairs.

The other portion was used to prepare the Illumina sequencing library using the TruSeq DNA Nano Library Prep Kit (Illumina, Inc., San Diego, CA, USA). Sequencing on the Illumina HiSeq X Ten platform using a 2 × 150 paired-end protocol yielded a total 7,614,332 reads and 1,149,764,132 total read bases. Quality filtering and adaptor trimming were performed using Trimmomatic v0.38 (6) ensuring a phred score of 30 or higher in 90% of the bases.

The HiFi reads were assembled *de novo* using HGAP v4 (7) to generate the draft genome assembly. Following this, Illumina reads were used to polish the genome with Pilon v1.21 (8) three times. The final draft genome of *Pseudomonas fluorescens* IMGN2 consisted of a single contig, totaling 6,727,739 base pairs, with an average GC content of 60.8% and an N50 value of 6,727,739. BUSCO v5.1.3 (9) analysis found it highly complete (99.19%).

The genome was annotated with the NCBI Prokaryotic Genome Annotation Pipeline (PGAP) (10), which yielded 6,130 genes, 6,044 coding sequences, 66 tRNA genes, and 16 rRNA genes. It was then compared to the Genome Taxonomy Database (GTDB) via GTDB-Tk v2.3.2 (11) and assigned to *Pseudomonas fluorescens*. Default parameters were used except where otherwise noted.

EggNOG annotation results showed proteins related to defense, secondary metabolite biosynthesis, and signal transduction. Its metabolic capabilities, including energy production and nutrient metabolism, support its role in plant health. The higher GC content of *Pseudomonas fluorescens* IMGN2 may influence its genomic properties though its implications for genomic stability require further research. This genome sequence offers prospects for sustainable agriculture, including microbial solutions for crop resilience and reduced chemical inputs.

## Data Availability

The annotated complete genome sequence has been deposited at the NCBI under the GenBank accession number CP151816. The BioProject database accession number is PRJNA1091830, and the BioSample accession number is SAMN40613482. The Sequence Read Archive information is available under the accession number SRR28580445 and SRR28580446.
